# Massive aggressive angiomyxoma of ischioanal region with relapse: A case report

**DOI:** 10.18502/ijrm.v22i4.16394

**Published:** 2024-06-12

**Authors:** Arezoo Naderzadeh, Amirhosein Attarbashi, Leila Pourali, Majid Ansari, Abbas Abdollahi

**Affiliations:** ^1^Endoscopic and Minimally Invasive Surgery Research Center, Mashhad University of Medical Sciences, Mashhad, Iran.; ^2^Supporting the Family and the Youth of Population Research Core, Department of Obstetrics and Gynecology, Faculty of Medicine, Mashhad University of Medical Sciences, Mashhad, Iran.

**Keywords:** Vulvar neoplasm, Perineum, Pelvic neoplasms, Recurrence.

## Abstract

**Background:**

Aggressive angiomyxoma (AA) is a rare and slow-growing tumor in the pelvic and perineal regions that might develop into other perineal structures. It can present variably, ranging from a painless mass to non-specific symptoms such as dyspareunia. Due to the high relapse rate, extensive tumoral resection is reasonably required to prevent recurrences. It is also commonly confused with other conditions such as lipomas, Bartholin's gland cysts, and hernias.

**Objective:**

A 43-yr-old female diagnosed with AA 10 yr ago was evaluated as a consequence of the tumor recurrence. She presented rare manifestations of a giant and cystic pelvic mass involving pararectal and paravaginal tissue in front of the sacrum.

**Case Presentation:**

Although AA is a rare and slow-growing tumor, close observation is recommended due to the high relapse rate. Furthermore, extensive tumoral resection and regular follow-up can reduce morbidity in these patients.

## 1. Introduction

Aggressive angiomyxoma (AA) is an uncommon and slow-growing vulvovaginal mesenchymal neoplasm categorized as an undifferentiated tumor based on the World Health Organization classification (1). Women experience a higher prevalence of the condition, with a ratio of 1 female to every 8.5 males affected (2). AA typically originates from the genital area in women of reproductive age. Although this tumor is known for its limited tendency to metastasize, it exhibits aggressiveness by infiltrating nearby structures (3).

The primary reasons for morbidity associated with this condition are recurrence and local invasion, which have reported in 35–72% of cases (4). AA usually appears as a vulvar polyp and is diagnosed histologically. It is also commonly confused with other conditions such as lipomas, Bartholin's gland cysts, and hernias (5, 6).

We herein discuss a rare case of AA in a 43-yr-old female with unusual recurrence involving the pararectal region that required extensive resection.

## 2. Case Presentation 

A 43-yr-old primiparous female came to the colorectal department of the Ghaem hospital, Mashhad, Iran, with a slow-growing large mass in the perineum around the rectum for 2 yr that caused the gluteal asymmetry. She had an ectopic pregnancy and in vitro fertilization around 11 yr ago. Her past medical history indicated that she had a mass on the left labia majora 10 yr ago and was diagnosed with a Bartholin cyst, for which she was treated conservatively. Due to the growth of the mass, computed tomography and MRI were done, and she underwent surgical removal of the mass through the perineum. The pathology of the lesion was AA. Then she underwent another surgery one year later, and the tumor was resected entirely since it had recurred. Considering the high recurrence rate, close follow-up was recommended, but after 2 yr, she voluntarily withdrew from the follow-up sessions.

She has had symptoms of gluteal asymmetry and a mass in her left area for the past 2 yr. In addition, she complained of severe vulvar discharge, constipation, and polyuria. On examination, a 5–10 cm mass was found in the left gluteal region, causing gluteal asymmetry.

On palpation, the mass was soft without tenderness. On inspection, no ulcer or visible discharge were observed. Rectal examination revealed an extra luminal pressure effect that was mobile. On vaginal examination, the effect of mass pressure was detected on the left lateral side of the vagina, but there was no lesion in the vagina or rectum.

**Figure 1:**
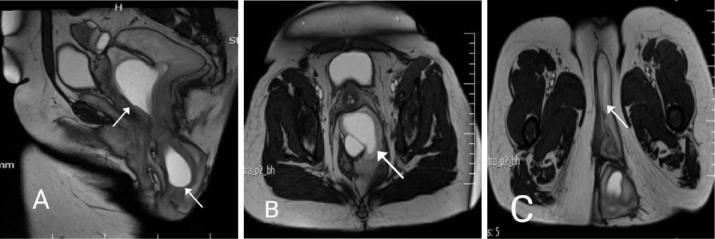
Pre-operation imaging A) Sagittal plane of pelvic MRI (T2-weighted MRI), indicating a large mass with longitudinal extension from the posterior of the uterus toward the ischiorectal and ischioanal spaces with a displacement of adjacent organs. B) Transverse plane of pelvic MRI indicates a left pararectal mass that causes displacement of the rectum to the right and is located behind the vagina. C) This MRI plane shows the involvement of the left side of the vagina (white arrow).

In routine laboratory investigations, severe anemia (Hb = 7.6 mg/dL) was detected, and a blood transfusion was administered. Abdominal and pelvic CT scans and a chest CT scan reported no abnormality or distant metastasis. Both the colonoscopy and the upper gastrointestinal endoscopy were normal. MRI revealed a gluteal mass with irregular, lobulated, and heterogenous borders situated posterior to the uterus, with the invasion of the soft tissue of the left perirectal and ischio-anal fossae. After injection, the mass had a heterogeneous enhancement and involved parts of the cervix and vagina. It was measured at approximately 65 mm × 70 mm, elongating to the urogenital fascia and deep soft tissue next to the left gluteus muscle and labia majora (Figure 1).

**Figure 2:**
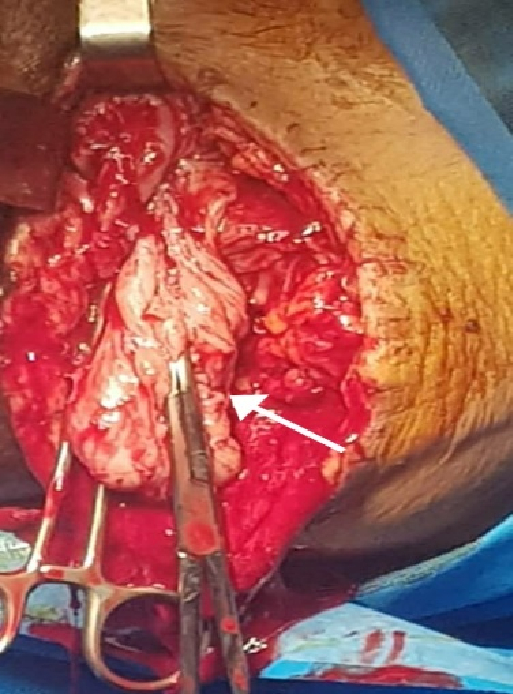
The white arrow (intra-operation field) indicates a left ischiorectal mass that extends the urogenital fascia upward.

Recurrent AA was the most suspected in the differential diagnosis of this mass, so she underwent extensive excision of the giant cystic tumor through the perineum. The tumor was located anterior and posterior to the sacrum, with severe adhesion to the surrounding tissue of the vagina and rectum. Therefore, resection with a safe margin was performed (Figure 2).

**Figure 3:**
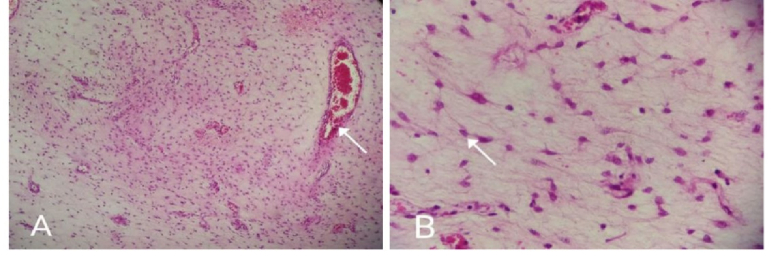
A) Histopathological findings indicate proliferation of vascular bundles. B) This figure shows spindle-shaped cells and myxoid stroma (white arrow).

Histopathological examination reported a mesenchymal neoplasm with extensive myxoid background and cellularity, consisting of bland cells and hyaline thick-walled vessels, consistent with AA (Figure 3). 4 days later, she developed a fever and a foul-smelling discharge from the wound. This caused her to return to the operating room again for a possible rectum injury. On reoperation, a rectal wall defect was observed where the mass had been removed. Subsequently, secondary healing of the rectum was successfully achieved through the next operations and colostomy insertion surgery. She was discharged in good condition, and the colostomy was closed after 4 months.

### Ethical considerations

Oral consent was obtained from the patient for the publication of this case report.

## 3. Discussion 

Our case report highlights a unique presentation of AA with initial occurrence at the left labia majora, followed by a late recurrence in the pelvic region posterior to the uterus, extending towards pararectal tissue and the vagina.

AA is a rare and slow-growing lesion that predominantly impacts the perineal and pelvic regions, mostly identified in women during their reproductive age (7). It can present variably, ranging from a painless mass next to the labia majora to non-specific symptoms such as dyspareunia, regional pain, and a feeling of pressure from the lump (5). According to the literature, the most commonly affected area is the vulva; however, the retroperitoneum and bladder can also be involved, less commonly (8). As in our patient-the first occurrence was at the left labia major and the second recurrence was at pelvic from the posterior of the uterus with extension toward pararectal tissue and vagina.

The tumor size varies from 1–60 cm (9). In our case, we reported the tumor size to be approximately 10 cm in the operation field. This tumor can be confused with vulvar masses such as vulvar lipoma and vulvar leiomyomatosis, vulvar abscess, Bartholin's gland cysts, Gartner duct cyst, vaginal polyp, vaginal prolapse, mesonephric duct cyst, vaginal hernia, instantaneous levator hernia, and other soft tissue neoplasms (5, 6). This locally invasive tumor might develop into other perineal structures like the vagina, rectum, and bladder (8). In our patient, the second recurrence involved the rectum and vagina.

Due to the high relapse rates with this tumor, extensive tumoral resection is reasonably required to prevent recurrences that occur in at least 30% of cases (10). Most recurrences happen within 5 yr following the primary surgery, with around 70% occurring in the first 3 yr. However, late recurrences as long as 14 yr after surgery have been reported (11), as our case had 2 recurrences after one year and 8 yr after the first resection of the tumor. In deep tumors, even resection of adjacent organs like the bladder or vagina may be considered (10). Likewise, our case underwent a partial and massive elimination of the rectum owing to the invasion of the tumor. Surgery is usually the mainstay treatment option for AA.

According to Wu et al., imaging modalities, including CT and MRI, play a crucial role in determining the extent of the tumor and locating the tumor before the operation, as there are complicated anatomical structures inside the pelvis (12). AA has many radiographic features. On CT, it demonstrates hypo- or isodense mass having a clear margin with less attenuation compared to muscle. Using enhanced CT or TI-weighted MRI, our report shows a swirled appearance with a tendency to invade other pelvic organs without muscle layer involvement. It is stated that both CT and MRI are diagnostic modalities. However, MRI with diffusion-weighted imaging has more specificity and superiority when it comes to establishing the precise location of the tumor and its adjacent structures (12). In our case, MRI revealed a gluteal mass with irregular, lobulated, and heterogeneous borders posterior to the uterus, with soft tissue invasion around the rectum. The mass had a heterogeneous enhancement after injection.

Histopathology and immunohistochemistry are used to make a definitive diagnosis of AA. There is a rubbery consistency, on the gross section of this tumor, and the gelatinous surface contains hemorrhagic points (9). Histologically, the tumor consists of stellate and spindle-shaped neoplastic cells, distinguished by a myxoid stroma. There is no evidence of mitosis, pleomorphism, or coagulation necrosis but abundant vascular malformation, including vessels of different sizes (13). In our case, histopathological evaluation reported spindle-shaped cells, myxoid stroma, and proliferation of vascular bundles consistent with AA.

Vimentin and CD34 are highly associated with this tumor, and desmin, estrogen receptor, and progesterone receptor are moderately linked to AA, but this tumor is negative for S-100 and CD68 (13). These findings indicate that AA will likely originate from mesenchymal cells with myofibroblastic and fibroblastic features. Based on the literature, AA usually has estrogen and progesterone receptors, which makes it reactive to gonadotropin-releasing hormone agonists (14). This can justify the high prevalence of this tumor among women of reproductive age. The first occurrence of AA in our patient occurred shortly after in vitro fertilization. Therefore, hormone suppression therapy was considered in our counseling sessions, and the patient underwent 6 months of hormone therapy with tamoxifen after the operations.

## 4. Conclusion

Since AA can be simply confused with other vulvar lesions like Bartholin's gland cysts, levator hernias, and other soft tissue neoplasms, any pelvic and vaginal mass among females must draw the attention to AA. However, pararectal recurrence is rare, and regular long-term follow-up can reduce morbidity in these patients.

##  Data availability

Data supporting the findings of this study are available upon reasonable request from the corresponding author.

##  Author contributions

A. Abdollahi and L. Pourali designed the study and conducted the research. A. Attarbashi, A. Naderzadeh, and M. Ansari monitored, evaluated, and analyzed the results of the study. Further, A. Attarbashi, A. Naderzadeh, and A. Abdollahi reviewed the article. All authors approved the final manuscript and take responsibility for the integrity of the data.

##  Conflict of Interest

The authors declare that there are no conflict of interest.
